# Stability and biosafety of human epidermal stem cell for wound repair: preclinical evaluation

**DOI:** 10.1186/s13287-022-03202-6

**Published:** 2023-01-05

**Authors:** Xiaohong Zhao, Xue Li, Ying Wang, Yicheng Guo, Yong Huang, Dalun Lv, Mingxing Lei, Shicang Yu, Gaoxing Luo, Rixing Zhan

**Affiliations:** 1 Institute of Burn Research, State Key Laboratory of Trauma, Burn and Combined Injury, Southwest Hospital, The Third Military Medical University (Army Medical University), Chongqing, 400038 China; 2 Department of Burn and Plastic Surgery, The First Affiliated Hospital of Wannan Medical College, Wuhu, 241001 Anhui China; 3 “111” Project Laboratory of Biomechanics and Tissue Repair, College of Bioengineering, Chongqing University, Chongqing, 400044 China; 4 Stem Cell and Regenerative Medicine, Southwest Hospital, The Third Military Medical University (Army Medical University), Chongqing, 400038 China

**Keywords:** Epidermal stem cells (EpiSCs), Wound repair, Biosafety, Preclinical

## Abstract

**Background:**

Cell therapy is a key technology to prevent sacrificing normal skin. Although some studies have shown the promise of human epidermal stem cells (EpiSCs), the efficacy, biosafety and quality control of EpiSC therapy have not been systematically reported.

**Methods:**

The biosafety, stemness maintenance and wound repair of EpiSC were systematically verified by in vitro and in vivo experiments. EpiSC were prepared from the foreskin using a collagen type IV rapid adherence method. The EpiSCs were identified by flow cytometry, immunofluorescence staining and cell morphology. The well-growing passage 1 (P1) EpiSCs were used to determine the proliferation curve (counting method). EpiSC clone formation assay was performed by Giemsa staining. Nude mice were used to prepare a full-thickness skin defect wound model to detect the repair effect of EpiSCs. The biosafety of EpiSCs was double tested in vitro and in vivo.

**Results:**

The results showed that the expression of specific markers and clone formation efficiency was stable when passage 1 (P1) to P8 cells were cultured, and the stemness rate of P8 cells was close to 85.1%. EpiSCs were expanded in vitro for 25 days, the number of cells reached 2.5 × 10^8^, and the transplantable area was approximately 75% of the total body surface area (TBSA). At 45 days, the total number of cells was approximately 30 billion, and the transplantable area was approximately the size of a volleyball court. A nude mouse wound model indicated that EpiSCs could rapidly close a wound. On postinjury day 7, the wound epithelialization rate in the cell transplantation group was significantly higher than that in the NaCl group (*P* < 0.05). In vitro, cell senescence increased, and telomerase activity decreased in P1 to P8 EpiSCs. In vivo, there were no solid tumors or metastatic tumors after EpiSC (P8) transplantation. In addition, the quality control of cultured cells met the clinical application criteria for cell therapy.

**Conclusion:**

This preclinical study showed the stability and biosafety of human EpiSC therapy for wound repair.

**Graphical Abstract:**

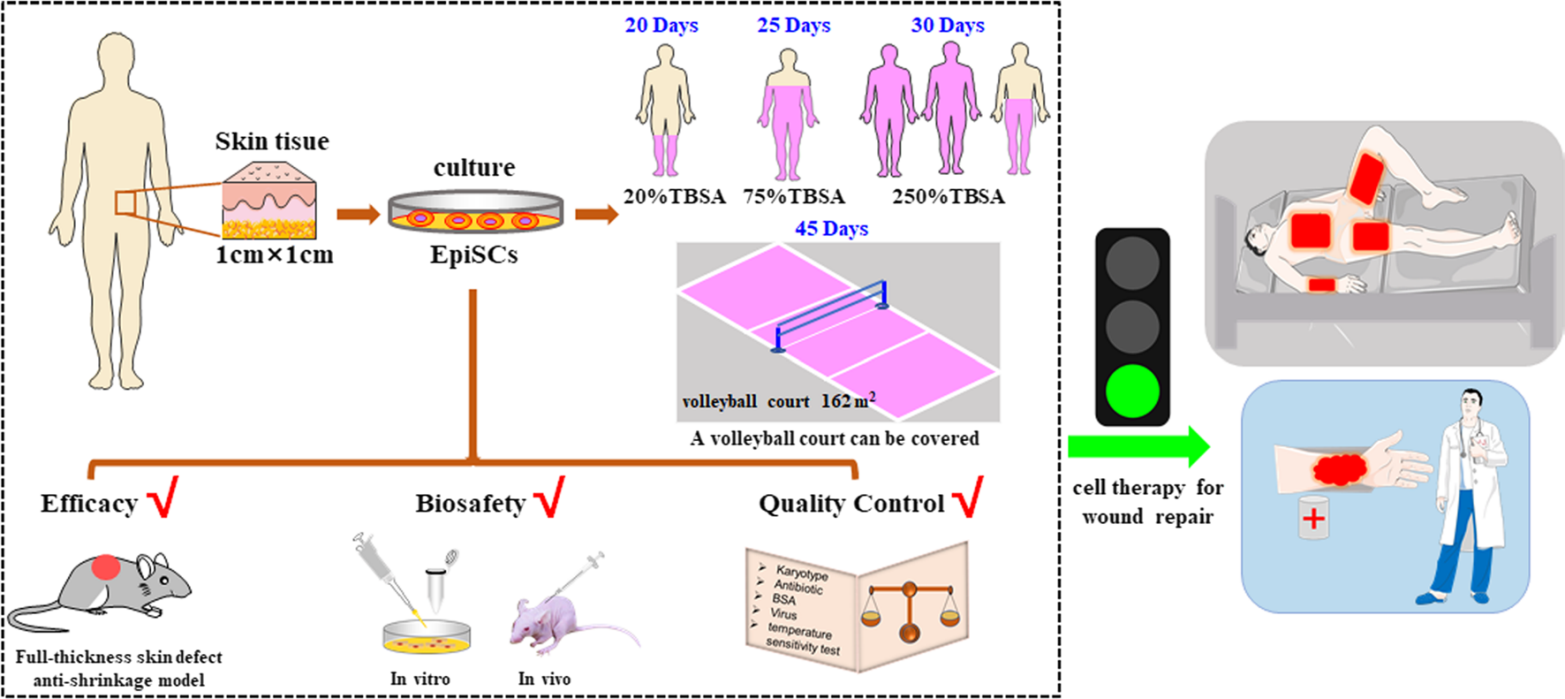

**Supplementary Information:**

The online version contains supplementary material available at 10.1186/s13287-022-03202-6.

## Introduction

Skin reconstruction techniques are used to repair large-area skin defects and treat diseases, such as junctional epidermolysis bullosa [1]. However, the reconstruction of skin after a severe burn is difficult due to skin shortages [2]. The current treatment methods mainly include microskin grafting, Meek microskin grafting, and flap transplantation [3, 4]. The traditional skin grafting strategy of “rob Peter to pay Paul” has three defects: (i) When repairing large skin defects, it is necessary to sacrifice normal skin as a skin source to repair the wound, (ii) in clinical treatment, multiple wound debridement surgical operations and grafting are often needed, and it takes several months to repair the wound, and (iii) the sacrifice of large-area normal skin usually leads to scar hyperplasia [5]. Therefore, how to avoid sacrificing normal skin and repair wounds in a timely and effective manner is the core issue in modern burn therapy.

Cultured epithelial cells have been used to improve the prognosis of patients with large-area skin defects and epidermolysis bullosa since 1981 [6, 7]. In cell therapy, the patient’s own epidermal cells are expanded in vitro, and epidermal replacement is performed by using noncultured autologous skin cells (the ReCell system) or a combination of cells with scaffolds and biomaterials [8]. The effect of autologous tissue-engineered skin in the treatment of large-area deep burn wounds and chronic ulcers is positive and similar to that of large autologous sectional skin grafts in reducing scar formation, improving function and appearance, and promoting other long-term outcomes [9, 10]. The characteristics of autologous tissue-engineered skin are mainly reflected in the construction of dermis and the selection of epidermal cells, in which the dermis cocultured with bovine collagen–glycosaminoglycan and autologous fibroblasts is the main studied autologous tissue-engineered composite skin. However, the selected epidermal cells are differentiated keratinocytes with weak proliferative ability in the process of wound remodeling [11]. Ensuring stable wound healing and epidermal regeneration after autologous tissue-engineered skin transplantation is a long-term concern in clinical treatment.

Epidermal stem cells (EpiSCs), which have strong self-renewal ability and the potential to produce highly differentiated progeny cells, are considered “seed cells” for skin rejuvenation [12]. Currently, clinical treatment with EpiSCs has been reported mainly for epidermolysis bullosa [7], vitiligo [13], Netherton syndrome, and burn wounds [14, 15]. These results indicate that EpiSCs hold promise for skin grafting without the need to sacrifice normal skin. In addition, EpiSCs have paracrine and autocrine activities, which can lead to the release of cytokines to regulate the tissue microenvironment. For example, some preclinical studies also showed that EpiSCs have the effect of anti-scarring in wound healing [16]. Therefore, these cells have wide application prospects in plastic surgery [17]. More importantly, EpiSCs are easily obtained from patients (scalp, dorsal skin, leg skin, toe gaps, etc.); thus, the use of these cells avoids the ethical issues associated with the use of fetal or embryonic tissue [12]. EpiSC therapy may become a new candidate therapy for burn wounds, but the effectiveness, biosafety, and quality control of its clinical application need to be further elucidated.

Therefore, a feasibility study of the clinical application of EpiSCs was conducted to establish a standard method for preparing autologous EpiSCs and to systematically evaluate the biosafety and effectiveness of expanding EpiSCs in vitro. The above studies further promote the clinical application of EpiSCs.

## Materials and methods

### Isolation, culture, identification, and proliferation of EpiSCs

#### Isolation and culture of EpiSCs

This study was approved by the Ethics Committee of the First Affiliated Hospital of Army Medical University. The batch number was (A) KY2021048. Based on previous research [18, 19], the method for preparing EpiSCs from the foreskin was briefly described below. After an operation, the prepuce tissue was soaked in 75% ethanol for 1 min and then washed with sterile PBS 3 times. Then, the subcutaneous tissue was removed and trimmed to 1 cm^2^. The tissue was digested with 0.25% neutral protease (Roche, Switzerland) at 4 °C for 12–16 h, and then, the epidermis and dermis were separated. The epidermis was shredded and digested with 0.25% trypsin (Sigma-Aldrich) at 37 °C for 15 min, and the digestion was terminated with 1640 medium without calcium (Thermo Scientific, MA) containing 10% fetal bovine serum (HyClone, UT). After filtration with a 70 μm membrane (Beyotime, China), the cell suspension was centrifuged at 800 rpm/min for 5 min, and the cell density was adjusted to 1 × 10^6^ cells/ml. The cells were inoculated into a culture flask embedded with collagen type IV (Sigma-Aldrich) and allowed to adhere for 10 min. Then, the nonadherent cells were carefully removed, and K-SFM medium (Thermo Scientific) with 10 μM Rho kinase inhibitor (Y-27632) (Sigma-Aldrich) was added for routine culture. The medium was changed to fresh medium without Y-27632 after 24 h. The culture medium containing Y-27632 on the first day of each passage was changed to medium without Y-27632 after 24 h.

#### Identification of EpiSCs

When the cell fusion ratio reached approximately 70–80%, the cells were digested with TrypLE™ Select (Thermo Scientific) and counted. The EpiSCs were identified by flow cytometry as follows: The cell density was adjusted to 1 × 10^6^ cells/ml, FITC-labeled mouse anti-human CD71 monoclonal antibody (Abcam, USA) single staining with 2 μl antibody was performed, PE-labeled rabbit anti-human CD49f monoclonal antibody (Abcam) single staining with 2 μl antibody was performed, and an incubation at 4 °C for 30 min was carried out. After that, the cells were detected by flow cytometry.

When the cell fusion ratio reached approximately 80%, the cells were digested with TrypLE™ Select and counted. The cells were inoculated with 1 × 10^5^ cells/ml into confocal plates that were pre-embedded with collagen type IV and routinely cultured for 2 days. Then, the medium was removed, 4% paraformaldehyde was added, and the cells were fixed for 15 min. After that, the cells were blocked with goat serum (Beyotime) at room temperature for 30 min. Next, the primary antibodies (mouse anti-human integrin β1 (1:500) (Abcam) and rabbit anti-human CK19 (1:500) (Beyotime)) were added and incubated overnight at 4 °C. Then, the secondary antibodies (Cy3-labeled goat anti-mouse IgG (H + L) (1:1000) and Alexa Fluor 488-labeled goat anti-rabbit IgG (H + L) (1:1000) (Beyotime)) were added and incubated at room temperature for 1 h. Finally, the cells were blocked with antifade mounting medium with DAPI (Beyotime), and laser confocal observation was performed.

#### Proliferation of EpiSCs in vitro

##### Determination of EpiSC proliferation curves

The well-growing passage 1 (P1) EpiSCs were used to determine the proliferation curve by direct counting with trypan blue staining during cell passage. The density of EpiSCs was adjusted to 1 × 10^6^ cells/ml and passed on to approximately 6000 cells/cm^2^. Three flasks were inoculated in each subculture, and one flask in each passage was used for the proliferation curve experiment. Subculturing was continued according to a previous method when the cell fusion ratio reached 70–80% (2.1.1). The cell count of each flask and the total number of cells were calculated according to the number of theoretical inoculation flasks. The cumulative cell area was calculated according to the number of flasks that could be inoculated after cell passage. The proportion of transplantable wound area (human total body surface area (TBSA) was 1.5 m^2^) was calculated according to the cumulative cultured cell area. Generally, the cells were passaged at a ratio of 1:4, and the cell fusion ratio was 70–80% after 4 days. The experiment was repeated three times.

##### EpiSC clone formation assay

Well-growing EpiSCs digested with TrypLE™ were selected to prepare a single-cell suspension. The cell density was adjusted to 5 × 10^5^ cells/ml. In all, 2000 cells were inoculated in a 12-well plate previously embedded with collagen type IV. The cells were cultured for 2 weeks. After that, the supernatant was removed and washed carefully with PBS twice. Then, 4% paraformaldehyde (Beyotime) was added for fixation for 15 min, the fixing solution was removed, Giemsa solution (Solarbio, China) was added for 10–30 min, and the dye solution was slowly washed off with tap water and dried in air. Finally, the clone formation rate was calculated. Clone formation rate = (number clones/number of inoculated cells) × 100%.

##### Analysis of specific markers for EpiSCs

Flow cytometry and immunofluorescence were used to detect the proportion of high expression CD49f, integrin β1, and CK19 and low expression CD71 in different cell passages. The methods were the same as before (2.1.2).

### EpiSCs repair full-thickness skin defect wounds in nude mice

#### EpiSCs repair full-thickness skin defect wounds

Twenty-four BALB/C nude mice (male) aged 7–8 weeks were purchased from the experimental animal center of Army Medical University, license number: SYXK (Chongqing) 20170002. The mice were randomly divided into the NaCl (saline) group (*n* = 12) and the EpiSC group (*n* = 12). The nude mice were anesthetized by intraperitoneal injection (7.5 ml/kg) of 1% pentobarbital sodium solution. Two full-thickness skin defect holes 6 mm in diameter were prepared on both sides of the backs of nude mice, and the two wounds of each mouse were PBS on the left side and EpiSCs on the right. The frequency of administration (local instillation) was once for the NaCl group (20 μl/well) and the EpiSC group (20,000 cells, 20 μl/well). All the wounds were closed with aseptic medical adhesive tape, and the nude mice were raised alone. The cells are described in Additional file 1: Materials and Methods (CM-Dil marks EpiSCs).

#### Observation of wound healing rate and sampling

On postinjury days (PIDs) 1, 3, 7, 11, and 15, wound healing was recorded with a digital camera. The wound healing rate was calculated by using ImageJ 1.52a image analysis software (National Institutes of Health). The nude mice were killed on PID 7 and 15 (three mice from each group). Skin tissue with a diameter of 1 cm was cut on the outer edge of the wound. Then, the skin samples were fixed with a 4% paraformaldehyde solution for 24 h. After that, they were dehydrated, embedded in paraffin, sliced (6 μm, the midline part of the wound healing skin was sectioned continuously and used for histological staining), and subjected to HE staining.

### Biosafety of EpiSCs

#### Senescence assay

The cell senescence assay was performed using a β-galactosidase staining kit (Beyotime). The P4, P8, and P9 EpiSCs were inoculated in a 6-well plate for 3 days, and then, the medium was absorbed and washed once with PBS. The subsequent steps were carried out according to the kit instructions. A detailed description of the materials and methods can be found in Additional file 1: Materials and Methods.

#### Telomerase activity assay

Telomerase activity was measured by using a telomerase reverse transcriptase (TERT) ELISA kit (BioVision, USA). First, the P1, P4, P8, and P9 EpiSC culture supernatants were prepared. Second, the supernatant was centrifuged at 1000 g at 2–8 °C for 20 min, and the supernatant was collected and stored at -20 °C. Finally, the follow-up operation was carried out according to the instructions.

#### Transcriptome analysis

For transcriptome analysis, P1, P4, P8, and P9 EpiSCs were incubated in RNAiso Plus (Takara) at a concentration of 1 × 10^6^ cells/ml and stored at − 80 °C. Three experimental replicates per group were performed. All the samples were transported to the Genomics Institute on dry ice for the transcriptome study. The Pearson correlation coefficients were based on all gene expression levels. A heatmap analysis of gene expression levels was created based on the averaged fragments per kilobase of exon per million fragments mapped (FPKM) values of genes in P1, P4, P8, and P9 EpiSCs. Genes with a fold change in expression > 1 and adjusted *P* value ≤ 0.001 were considered to be differentially expressed genes (DEGs). The selected DEGs related to tumorigenicity, proliferation, and aging were analyzed. A detailed description of the materials and methods can be found in Additional file 1: Materials and Methods.

#### Tumor experiments

Six-week-old female athymic nude mice (all purchased from the experimental animal center of Army Medical University, license number: SYXK (Chongqing) 20170002) were randomly divided into 3 groups: the NaCl group (*n* = 10), the B16 cell group (*n* = 10), and the EpiSC group (*n* = 10). The nude mice were treated subcutaneously on the back. The NaCl group was subcutaneously injected with 200 μl NaCl. The B16 group was subcutaneously injected with 200 μl NaCl with 1 × 10^6^ B16 cells. In the EpiSC group, 200 μl NaCl with 1 × 10^7^ EpiSCs (P8) was injected subcutaneously. The B16 cell group was used as the positive control group (thought to be able to cause tumor formation). The nude mice were kept in standard SPF rooms, and the experiments complied with ethical norms and animal welfare requirements. The growth of the tumor was observed and recorded every 3 days. When the tumor grew large enough to prevent the nude mice from moving, the mice were killed immediately, and samples (heart, liver, spleen, lung, kidney, and skin tissue of the transplant site) were collected. When the EpiSC transplantation group reached 4 months, all the nude mice were killed, and samples (heart, liver, spleen, lung, kidney, and skin tissue of the transplant site) were collected for follow-up observation.

#### Histological analysis

The foreskin sample and nude mouse tissues were collected and fixed with 4% paraformaldehyde (Beyotime) for 24 h. The tissues were paraffin-embedded after dehydration. The slice thickness was 6 μm, and the sections were stained with hematoxylin–eosin.

### Statistical analysis

All the data are expressed as the mean ± SD. Statistical analysis was performed by using GraphPad Prism 5 software (GraphPad Software Inc., CA, USA). One-way ANOVA and the Tukey/Dunn test were used to assess multiple comparisons. A *P* value < 0.05 was considered statistically significant.

## Results

### Separation, culture, identification, and amplification of EpiSCs

#### Separation, culture, and identification of EpiSCs

**Fig. 1:**
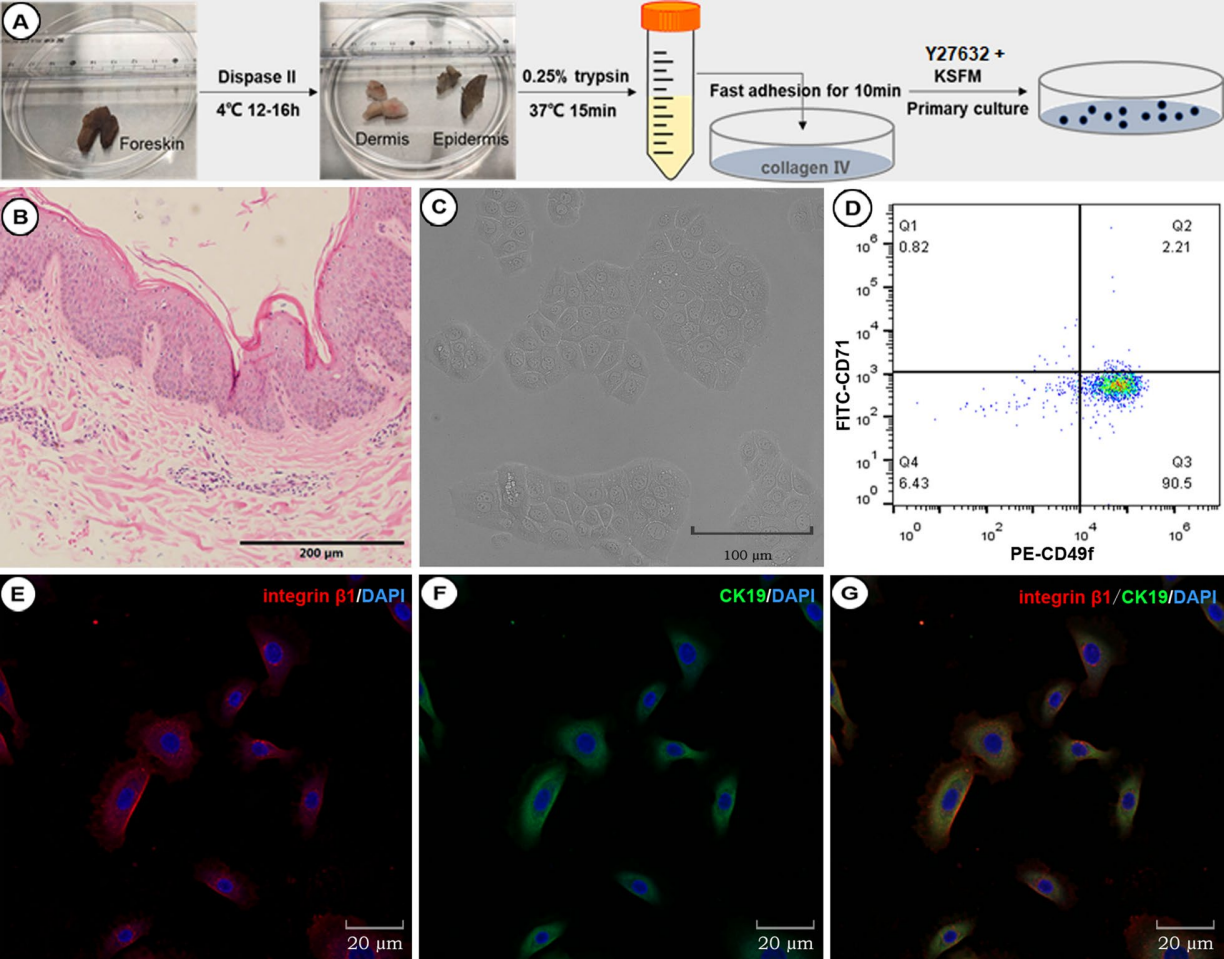
Preparation and identification of EpiSCs from the human prepuce. **A** The method of preparing EpiSCs from the human prepuce. **B** HE staining results of the prepuce (under 10 × optical microscopy, the scale was 200 μm). **C** Morphology of cultured cells (P1) (under 20 × optical microscopy). **D** Flow cytometry detection of cultured cells (P1). Q3 indicated that the ratio of high CD49f expression to low CD71 expression was approximately 90.5%. (E, F, G) Immunofluorescence staining of cells (P1). Blue indicates DAPI, red indicates integrin β1 (Cy3), and green indicates CK19 (Fluor488) (under 40 × confocal microscopy)

The stable expansion of EpiSCs is a prerequisite for cell transplantation-related surgery. The method for preparing EpiSCs was carried out as follows (Fig. 1A). A detailed description of the materials and methods can be found in “Isolation and culture of EpiSCs” section. The foreskin structure included the epidermis and dermis, and the epidermis had a large number of cells restained with hematoxylin (Fig. 1B). It has been reported that EpiSCs can be identified based mainly on the combination of CD49f, CK19, integrin β1, and CD71 [12]. The epidermis was isolated and processed into a single-cell suspension, and flow cytometry showed that the proportion of cells with high CD49f expression and low CD71 expression was approximately 9% (Additional file 1: Fig. S1). The cells had typical paving stone and clonal formation (Fig. 1C). Flow cytometry showed that the proportion of cells with high CD49f expression and low CD71 expression was 90.5% (Fig. 1D). Immune fluorescence staining showed that the cultured cells expressed high levels of integrin β1 and CK19 (Fig. 1E–G). Therefore, the method can be used to stably isolate and prepare EpiSCs in vitro.

#### Rapid amplification capacity of EpiSCs in vitro

**Fig. 2:**
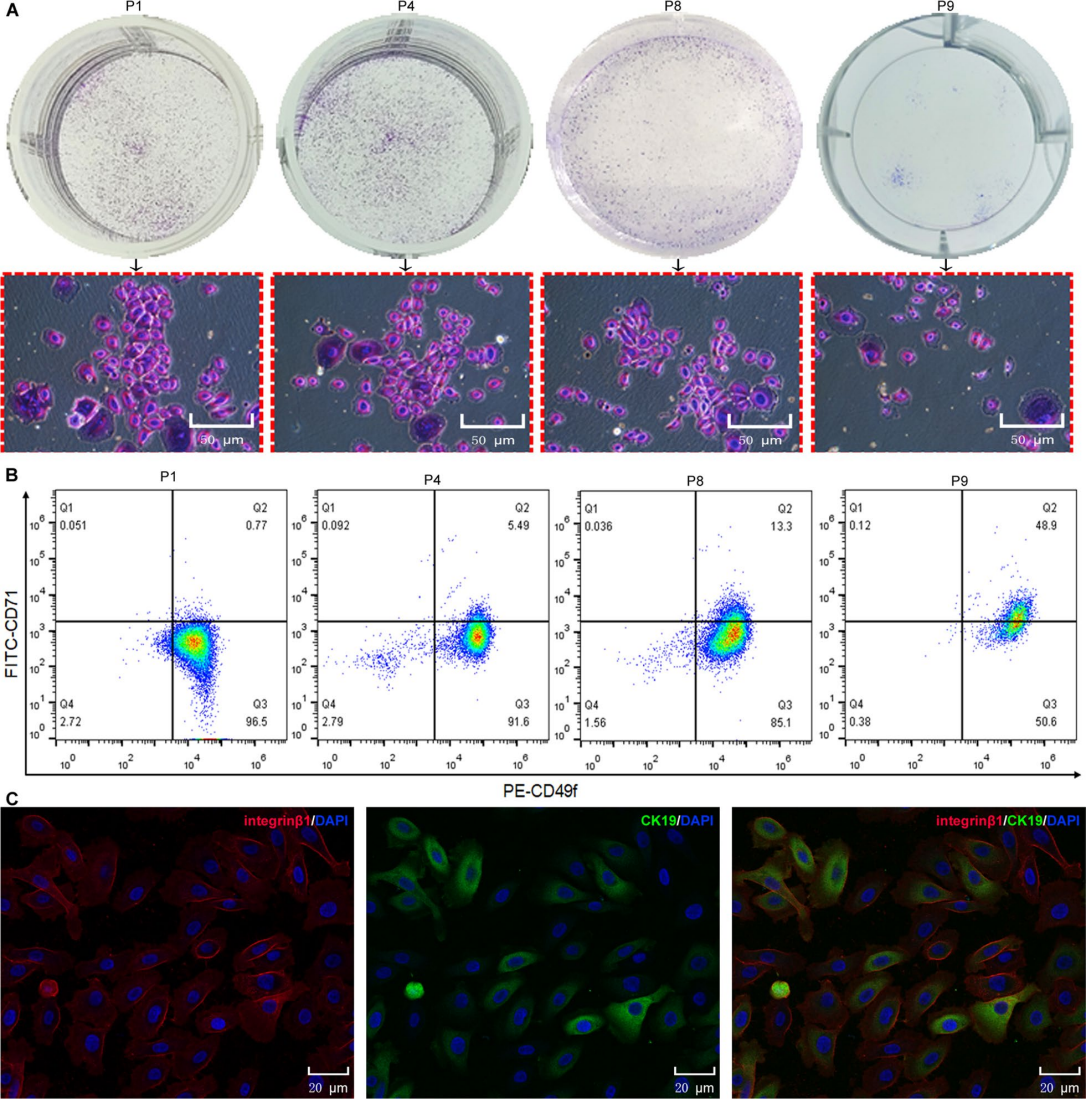
Clone formation and “stemness” of EpiSCs were analyzed. **A** Clone formation of different EpiSC passages. The red block diagram represents the enlarged picture directly above. **B** Flow cytometry analysis of the proportion of CD49f and CD71 expression in different cell passages. **C** Immune fluorescence staining of P8 cells. Blue indicates DAPI, green indicates CK19 (Fluor 488), and red indicates integrin β1 (Cy3). The experiment was repeated three times

To assess whether EpiSCs meet the need for severe skin defect wound transplantation, we investigated the colony formation, “stemness” maintenance, and proliferation curve of EpiSCs in vitro. The clone formation rate reflects the two important traits of cell population dependence and proliferation ability. We observed obvious cell clones in P1, P4, and P8; the cloning rate of P1 cells was approximately 31.7%, that of P4 cells was approximately 33.1%, and that of P8 cells was approximately 21.7%, but almost no clones were observed in P9 cells (Fig. 2A). Flow cytometry was used to detect the expression of CD49f and CD71 in different passages. When cells were cultured to P8, more than 85.1% of the cells still expressed high CD49f and low CD71 levels (Fig. 2B). Immune fluorescence staining showed that the P8 cells expressed high levels of CK19 and integrin β1 (Fig. 2C). In summary, the proliferation ability and “stemness” of EpiSCs expanded in vitro remained stable.

**Fig. 3:**
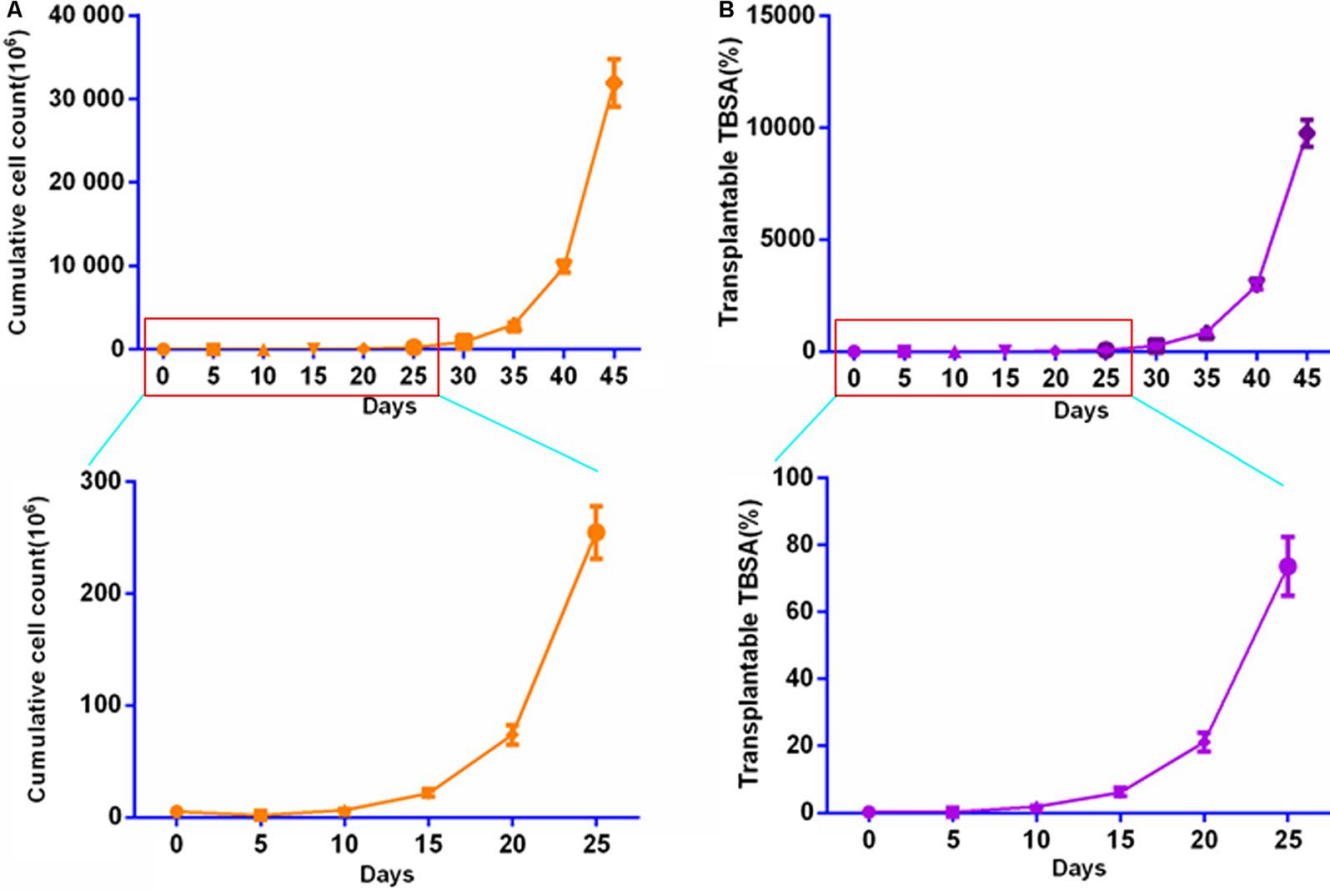
Proliferation curve of EpiSCs from the human prepuce. **A** Proliferation of EpiSCs in vitro. **B** EpiSC transplantable wound area ratio. Note. Isolated and cultured prepuce EpiSCs from males of ages 6, 14, and 18 years. The area of each prepuce was approximately 1 cm^2^. The cumulative cell area was calculated according to the number of flasks that could be inoculated after cell passage. The proportion of transplantable wound area (the TBSA was counted according to 1.5 m^2^) was calculated according to the cumulative cultured cell area

Approximately 6 × 10^6^ skin cells were dissociated from the 1 cm^2^ foreskin. The skin cell suspension was inoculated into two T25 culture flasks and then purified and cultured to amplify EpiSCs. When P0 cell fusion reached approximately 70%, the cells were passaged according to 5 × 10^5^/T75 flask. When the cells were cultured to P3, the cumulative cell count was 73.93 ± 7.15 million, the expansion time was approximately 20 ± 2 days, and the transplantable wound area (%) was 21.17 ± 2.32. When the cells were cultured for 30 ± 2 days (P5), the expanded cell number and the transplantable wound area were sufficient for more than 90% of the wounds in duplicate experiments (Fig. 3 and Additional file 1: Table S1).

### EpiSCs repair full-thickness skin defect wounds in nude mice

**Fig. 4:**
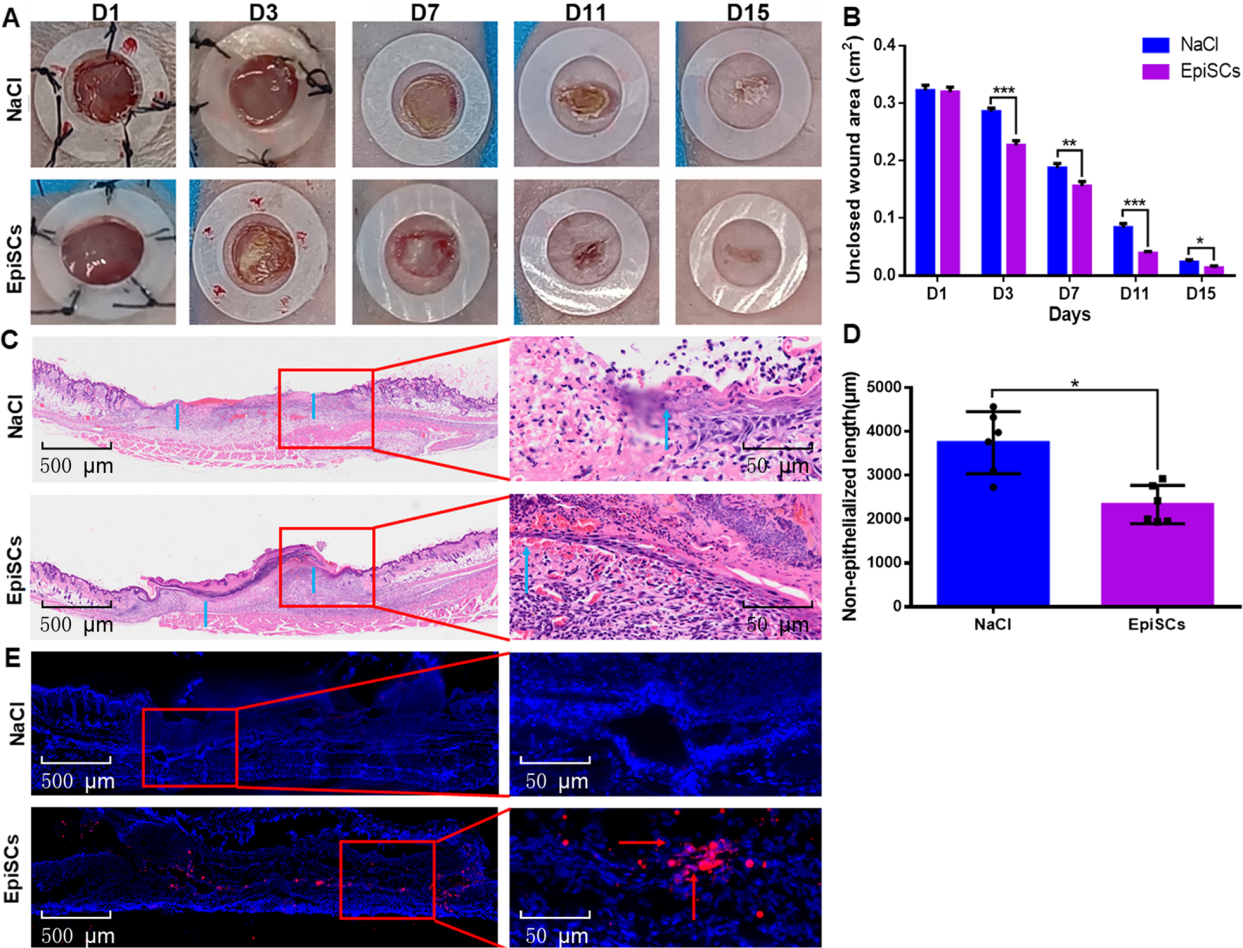
EpiSCs repair full-thickness skin defect wounds in nude mice. **A** Digital cameras recorded the size of skin wounds at different time points. **B** Statistical results of the unclosed wound area in the NaCl group and the EpiSC group at different time points (*n* = 6). **C** On PID 7, the results of HE staining in each group. The red box is the epithelialization location, and the blue arrows indicate the epithelialization site of the wound. **D** The statistical results of the length of the skin wound without epithelialization (*n* = 6). **E** On PID 7, EpiSC transplantation was performed in immunodeficient mice, and the red arrows indicate transplanted cells. The EpiSC group vs. the NaCl group, *P* < 0.05

A fundamental problem of wound cell therapy is how to ensure the survival of transplanted cells, so this study was designed to not only observe the effect of EpiSCs on wound repair but also demonstrate that transplanted cells can survive in new wounds for a long time. On PIDs 1, 3, 7, 11, and 15, wound healing was observed, and the unclosed wound area was counted (Fig. 4A and B). On PID 1, the wound area size was not different, and a full-thickness skin defect with a diameter of 6 mm was visible. On PIDs 7, 11, and 15, the unclosed wound area of the EpiSC group was significantly smaller than that of the NaCl group (*P* < 0.01). On PID 7, the wounds were collected for HE staining. The re-epithelialization rate of the EpiSC group was significantly faster than that of the NaCl group (*P* < 0.05) (Fig. 4C and D). In addition, CM-DIL-labeled EpiSCs were stained bright red (Additional file 1: Fig. S2). On PID 7, the CM-DIL-labeled EpiSCs survived at the junction of the epidermis and dermis (Fig. 4E).

### Biosafety of EpiSCs

#### Biosafety of EpiSCs in vitro

**Fig. 5:**
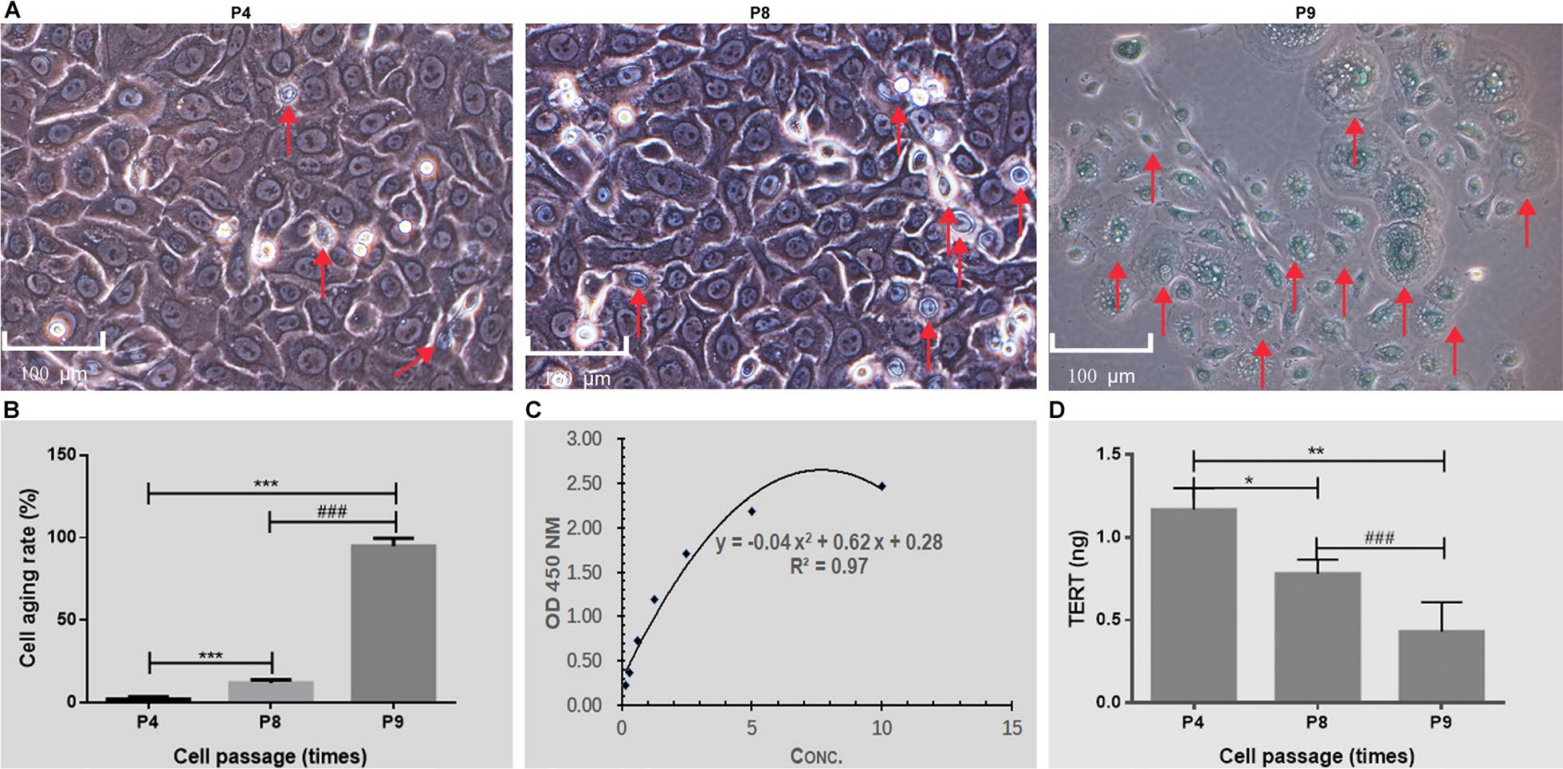
Biosafety of EpiSCs in vitro. **A** β-Galactosidase senescence staining of EpiSCs (P4, P8, P9). **B** Cell senescence staining statistics. Six visual fields were counted in each group. **C** Standard curve for detection of telomerase activity. **D** Detection of telomerase in EpiSC culture supernatant, P8 vs. P4, *P* < 0.01; P9 vs. P4, *P* < 0.01; P9 vs. P8, *P* < 0.01. (*n* = 5). Note. The red arrow indicates senescent cells (senescent cells were dyed blue). Concentration (Conc.)

Cell senescence is considered a process by which tumors are inhibited in organisms, and it is also a potential cause of biological aging [20]. Senescent cells usually become larger and express β-galactosidase with high enzyme activity at pH 6.0 [21]. In this study, there were fewer senescent cells among the P4 cells. However, the P8 cells showed an obvious trend of senescence, and the P9 cells were completely senescent (Fig. 5A). The results of further statistical staining showed that the senescent proportion of P8 cells was significantly higher than that of P4 cells (*P* < 0.01), and P9 cells showed approximately 100% senescence (Fig. 5B). Telomerase activation plays a very important role in the balance between aging and tumors. The determination of telomerase activity in EpiSC culture supernatant indicated that the content of TERT in P9 cells was lower than that in P8 cells (*P* < 0.01), and the content of TERT in P8 cells was lower than that in P4 cells (*P* < 0.05) (Fig. 5D).

#### mRNA expression of different EpiSC passages

**Fig. 6:**
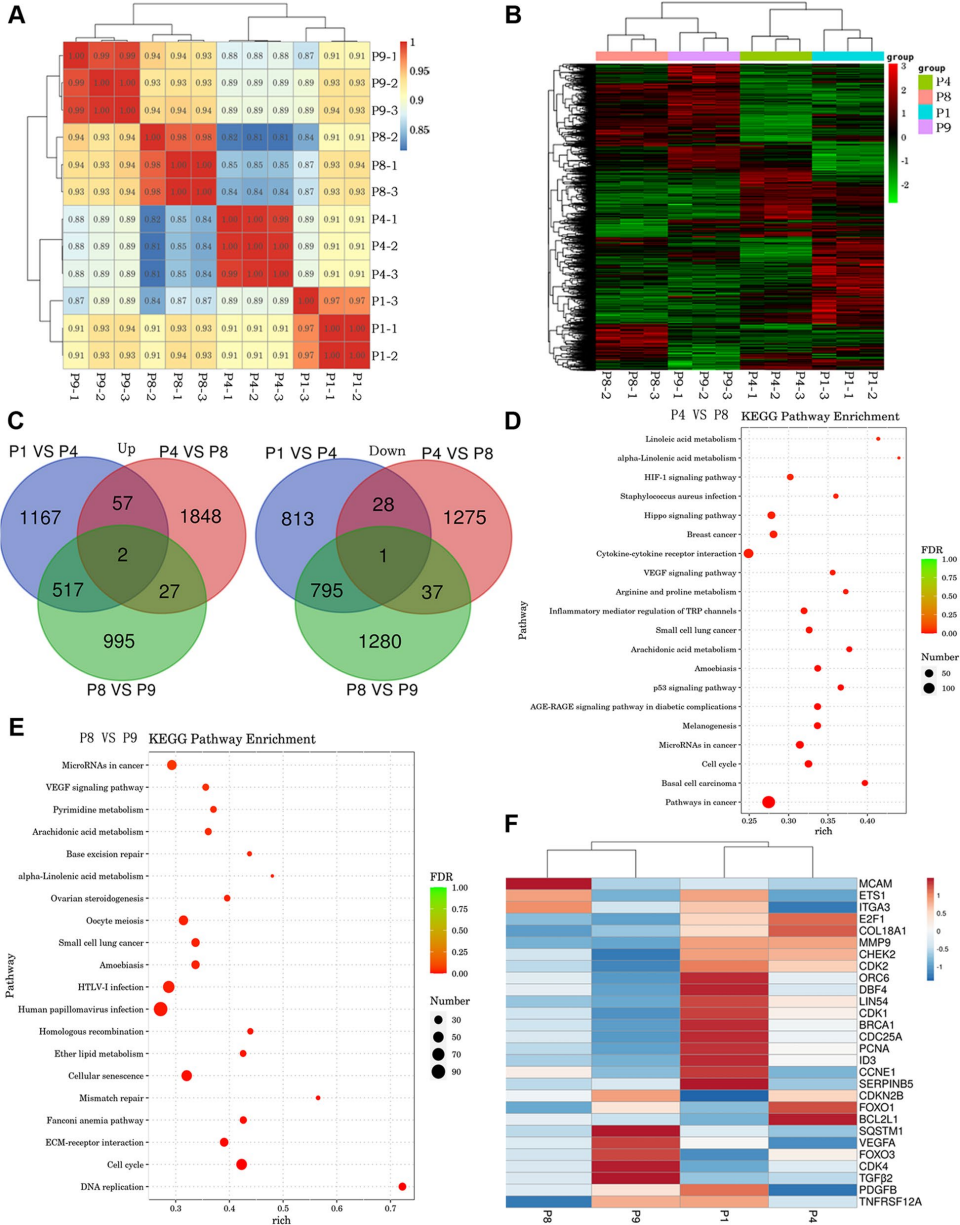
mRNA sequencing results of EpiSCs. **A** Sample correlation test of the P1, P4, P8, and P9 EpiSCs. **B** DEG clustering of the P1, P4, P8, and P9 EpiSCs. **C** Relationship between the upregulated and downregulated DEGs in different groups. **D** KEGG functional classification of the DEGs (P4 vs. P8). **F** KEGG functional classification of the DEGs (P8 vs. P9). **F** The expression heatmap of representative genes related to cell proliferation, senescence, and tumorigenicity in each group (*n* = 3)

To understand the transcriptional responses of EpiSC subculture, we performed mRNA sequencing (mRNA-seq) analysis of EpiSCs at P1, P4, P8, and P9. The Pearson coefficient showed that the gene expression similarity was very high (correlation coefficient > 0.8) between the P1, P4, P8, and P9 samples (Fig. 6A). Cluster analysis of the DEGs showed that the genes related to expression between the P1 and P4 samples were grouped and that those between the P8 and P9 samples were grouped (Fig. 6B). The relationship between the upregulated and downregulated DEGs was different between the groups (Fig. 6C). There were 59 upregulated genes and 29 downregulated genes in the P1 vs. P4 samples and the P4 vs. P8 samples, respectively. There were 29 upregulated genes and 38 downregulated genes in the P4 vs. P8 samples and the P8 vs. P9 samples, respectively. To further investigate the responses of EpiSC subculture to cooperative signaling, KEGG enrichment analysis was performed to identify specific biological processes enriched among the significant DEGs. These pathways included arachidonic acid metabolism, cellular senescence, the VEGF signaling pathway, ECM-receptor interactions, etc. In the P4 vs. P8 and P8 vs. P9 samples, all of these pathways were crucial for regulating lipid metabolism, insulin sensitivity, and cellular processes (Fig. 6D and E).

In addition, compared with the P1 group, the P9 group exhibited 1711 upregulated genes and 1782 downregulated genes. There were 1753 upregulated genes and 1014 downregulated genes in the P8 group. There were 1743 upregulated genes and 1643 downregulated genes in the P4 group (Additional file 1: Fig. S3A). KEGG enrichment analysis showed that the pathways included the PI3K-AKT, MAPK, and Wnt VEGF signaling pathways (Additional file 1: Fig. S3B). Gene expression associated with proliferation, aging, and tumorigenesis was further analyzed. The results of statistical sequencing showed that with increasing passages, proliferation-related genes (PCNA, CHEK2, CDK2, DBF4, BRCA1, and LIN54) were downregulated, senescence genes (BCL2L1, CDK4, Foxo3, CDKN2B, and ETS1) were upregulated, and tumorigenicity-related genes (COL18A1, MMP9, MCAN, and ITGA3) were downregulated (Fig. 6F). In summary, cell proliferation decreased and senescence increased with increasing passages.

#### Tumorigenicity of EpiSC transplantation in vivo

**Fig. 7:**
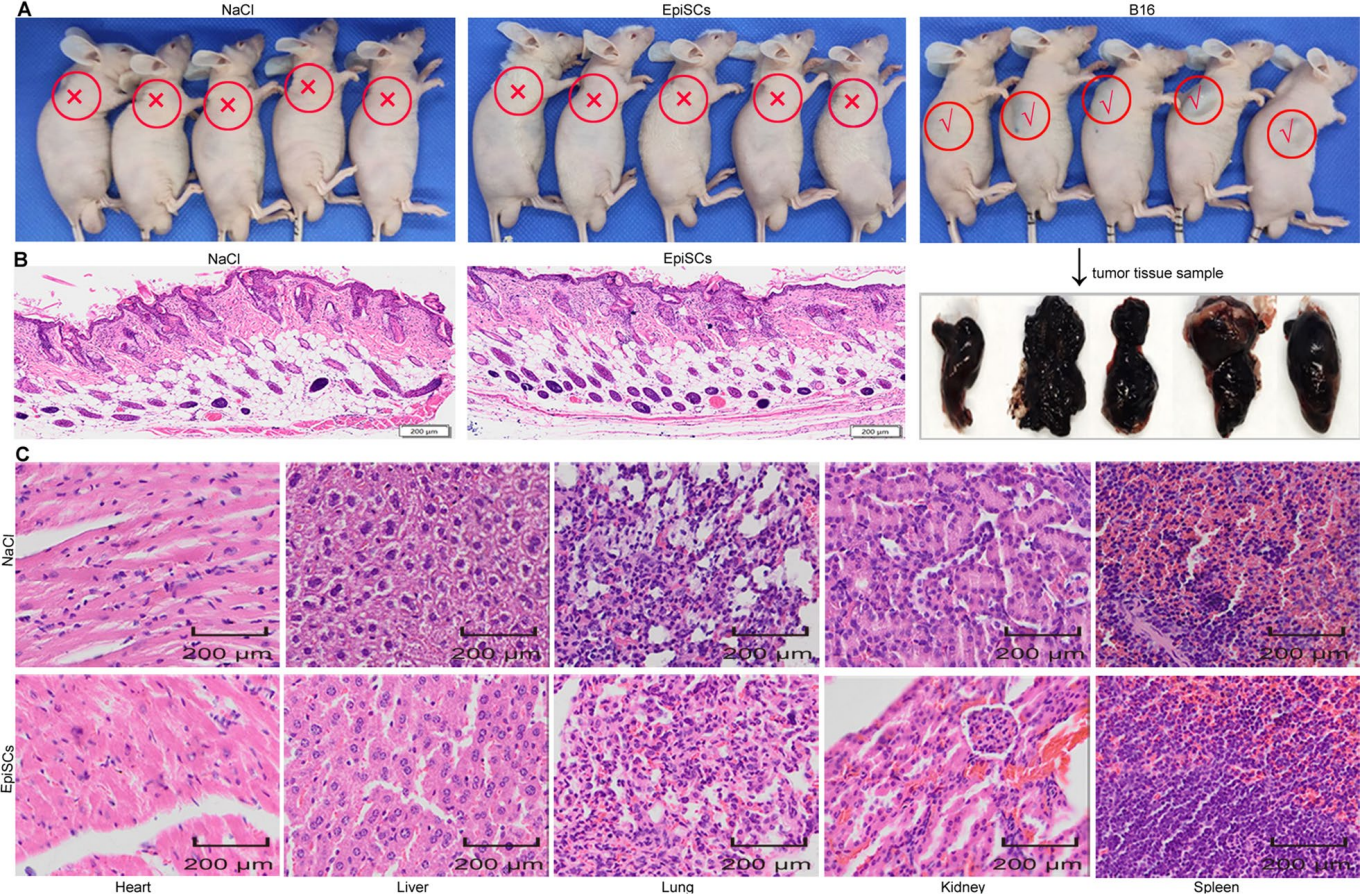
Tumorigenicity of subcutaneously injected EpiSCs in vivo. **A** Normal saline, EpiSCs, and B16 cells were injected subcutaneously into nude mice. “✕” indicates that there was no tumorigenesis 4 months after normal saline and EpiSC transplantation, and “✓” represents tumor formation on the 12th day after B16 transplantation. **B** Four months after the operation, the skin of the subcutaneous injection site was stained with HE in the NaCl group and the EpiSC group. **C** Four months after the operation, the heart, liver, spleen, lung, and kidney were stained with HE in the NaCl group and EpiSC group. Note. The subcutaneous injection site is marked by the red circle, “✕” means no tumor formation, and “✓” means tumor formation

Tumorigenicity refers to the process of tumor formation in animals after subcutaneous injection of the cells to be tested. The purpose of this assessment is to determine the ability of the cell matrix to form tumors after transplantation. As shown in Fig. 7A, neither the NaCl group nor the EpiSC group had tumor formation on the anterior dorsal side 4 months after the operation, and the nude mice remained healthy. However, the B16 group grew obvious solid melanoma tissue on the 12th day after surgery. In addition, the organs (heart, liver, spleen, lung, kidney) of the nude mice in the saline group and the EpiSC group were brightly colored 4 months after the operation, and there was no tumor-like growth on the surface (Additional file 1: Fig. S4).

HE staining was used to analyze subcutaneous injection sites and organ tumorigenesis in the NaCl group and EpiSC group 4 months postoperatively. As shown in Fig. 7B, there was no abnormality in the subcutaneous injection site of the EpiSC group compared with the normal saline group, and both groups showed healthy skin structures. In addition, there were no tumor-like cells in the heart, liver, spleen, lung, or kidney in any of the groups (Fig. 7C).

## Discussion

EpiSCs have a history of more than 40 years from discovery to clinical application [22]. Currently, the reported methods of isolating EpiSCs involve the use of type I collagen, type IV collagen, fibronectin, laminin rapid adherence, etc. However, researchers usually use the type IV collagen rapid adhesion method to isolate EpiSCs [19]. Although this method can be used to steadily expand primary cells, individual differences lead to senescence of cultured cells, affecting the purity (stemness), and function of EpiSCs. The “stemness” of EpiSCs after amplification has not been reported. Our results showed that when expanded to P8, EpiSCs showed stable “stemness” and clone formation efficiency (Fig. 2). Transcriptome sequencing showed that the gene expression in the cells was relatively stable (Fig. 6).

Although Howard Green and his colleagues have already effectively demonstrated and utilized keratinocyte colonies to treat burn patients since the 1980s, no systematic analysis of the long-term survival and distribution of EpiSCs in promoting wound repair has been reported [23, 24, 25]. In clinical practice, prepassage cells are usually used to reduce the risk of transplantation. We transplanted P5 cells into full-thickness skin defect wounds to evaluate the repair function of EpiSCs. The results showed that EpiSCs promoted wound epithelialization and that EpiSCs existed for a long time at the junction of the dermis and epidermis in reconstructed tissue (Fig. 4). These findings suggest that the transplanted EpiSCs are functional cells of skin regeneration and remodeling in the wound microenvironment. In addition, mesenchymal stem cells (MSCs) play an important role in wound repair. It has been reported that MSCs can differentiate into keratinocytes and fibroblasts to regulate the damaged tissue [26]. Umbilical Cord MSCs secrete anti-inflammatory factors to reduce the level of wound inflammation and increase the rate of wound healing [27]. Exosomes released from educated bone marrow MSCs accelerate cutaneous wound healing via promoting angiogenesis [28, 29]. Therefore, EpiSCs can be combined with MSCs to regulate wound healing. Skin grafting is mainly a strategy for treating large-area skin defects, but it is very difficult to treat patients with insufficient skin sources. EpiSCs have been reported to be stable during in vitro subculture, but no specific experimental data have been reported to verify this finding [30]. Therefore, we explored the feasibility of rapidly closing large-area skin defects with EpiSCs cultured in vitro. The prepuce EpiSCs from donors were expanded in vitro for 45 days, the total number of cells was approximately 30 billion, and the transplantable area was approximately the size of a volleyball court. In clinical application, these cells can withstand 20 failed operations or maintain a minimum 90% cell survival rate and wound closure rate. The body surface area was 2.0 m^2^ (international standard).

Similar to other cell therapies, the most important issue in the clinical use of EpiSCs is biosafety [31]. In clinical studies, EpiSCs cultured in vitro have not been reported to cause biosafety problems, but this does not mean that these cells are safe. Recently, the safety concerns of scholars have mainly involved in vitro residues and tumorigenicity in vivo [32, 33, 34]. Tumorigenicity mainly focuses on cell telomerase activity, cell senescence, and tumor formation after transplantation in vivo [35, 36, 37]. Telomere length is gradually shortened with the incomplete replication of chromosomes. When telomerase activity decreases, the telomere shortens, so telomerase activation plays a very important role in the balance between aging and tumors [38, 39]. The vast majority of normal cells are considered to have limited mitotic capacity and enter a state of senescence after being unable to divide [40]. Here, we explored the biosafety of EpiSCs from two aspects: culture (in vitro) and transplantation (in vivo). In vitro culture, our results showed that with increasing passages, EpiSCs gradually became senescent, telomerase activity decreased, the expression of cell proliferation-related genes decreased, the expression of senescence genes increased, and the expression of tumorigenicity-related genes decreased, indicating that EpiSCs cultured in vitro showed normal mitosis and there was no risk of tumor formation. The transplantation experiment in immunodeficient mice showed that our transplanted cells did not produce solid tumors or metastatic tumors at 4 months. In this study, P9 cells showed more obvious senescence than P8 cells. From the sequencing results, it was inferred that this is related to the significant activation of cell senescence-related pathways. In principle, autologous cell transplantation will not produce immune rejection, but autologous cell expansion in vitro due to long-term exposure to the culture environment increases the risk of immune rejection. Therefore, the residues mainly detected were antibiotics, BSA, cytokines, etc. (Additional file 1: Quality control data, Figures S5–S9 and Table S2) [41]. The results showed that the quality control of cultured cells met the clinical application criteria of cell therapy. These experiments confirmed that the EpiSCs cultured with our improved scheme were relatively safe. Nevertheless, we suggest that the safety of cell therapy requires clinical reconfirmation and long-term follow-up.

This study only demonstrated the wound repair function, cell safety, and quality control of EpiSCs at the cellular and animal levels. How to effectively improve the traumatic microenvironment and low survival rate of transplanted cells is an urgent cell therapy problem to be solved [42]. Autologous tissue-engineered composite skin is closest to normal skin in tissue structure and physiological function, so we are considering the use of autologous EpiSCs to construct tissue-engineered composite skin in future research. In addition, the biosafety of cells should be followed up for a long time in the course of clinical treatment.

## Conclusions

In summary, we achieved rapid expansion and “stemness” maintenance of EpiSCs by a modified method. We elucidated the biosafety, effectiveness, and quality control of these cells at the cellular and animal levels for the first time. This study lays the groundwork and guidance for the closure of burn wounds and the treatment of skin defects and for the construction of tissue-engineered skin and its clinical application. In the next study, clinical trials will be conducted to explore the survival rate of the cells and observe the safety of cell transplantation.

## Supplementary Information

**Additional file 1: Materials and Methods. Fig. S1.** Identification of EpiSCs from human prepuce. **Fig. S2.** EpiSCs (P4) were stained with CM-Dil. **Fig. S3.** The mRNA sequencing results of EpiSCs. (A) Relationship between up-regulated and down-regulated genes of DEGs in different groups. (B) KEGG functional classification of the DEGs. **Fig. S4.** The organs of the NaCl group and the EpiSC group. **Table S1.** The proliferation curve of EpiSCs. **Quality control data** (**Fig. S5.** Statistical results of cell viability before and after cryopreservation. **Fig. S6.** Effect of high temperature on epidermal cells. **Fig. S7.** Effect of low temperature on epidermal cells. **Fig. S8.** Effect of long-term experiment on epidermal cells. **Fig. S9.** Detection of CD73, CD90, and CD105 expression in EpiSCs (P8) by flow cytometry. **Table S2.** Items and standards for testing the quality of EpiSCs.

## Data Availability

The data that support the findings of this study are available from the corresponding author upon reasonable request.

## Abbreviations

EpiSCs: Epidermal stem cells
P1: Passage 1
TBSA: Total body surface area
PIDs: Postinjury days
TERT: Telomerase reverse transcriptase
DEGs: Differentially expressed genes
MSCs: Mesenchymal stem cells
