# Supplementary material for: Stability and biosafety of human epidermal stem cell for wound repair: preclinical evaluation
Source: Stem Cell Res Ther. 2023 Jan 5;14:4. doi: 10.1186/s13287-022-03202-6 (PMC9814209; doi:10.1186/s13287-022-03202-6)
Supplement: Supplementary file 1 — Additional file 1: Materials and Methods. Fig. S1. Identification of EpiSCs from human prepuce. Fig. S2. EpiSCs (P4) were stained with CM-Dil. Fig. S3. The mRNA sequencing results of EpiSCs. (A) Relationship between up-regulated and down-regulated genes of DEGs in different groups. (B) KEGG functional classification of the DEGs. Fig. S4. The organs of the NaCl group and the EpiSC group. Table S1. The proliferation curve of EpiSCs. Quality control data (Fig. S5. Statistical results of cell viability before and after cryopreservation. Fig. S6. Effect of high temperature on epidermal cells. Fig. S7. Effect of low temperature on epidermal cells. Fig. S8. Effect of long-term experiment on epidermal cells. Fig. S9. Detection of CD73, CD90, and CD105 expression in EpiSCs (P8) by flow cytometry. Table S2. Items and standards for testing the quality of EpiSCs. [file 13287_2022_3202_MOESM1_ESM.doc]

**Additional file 1**

Stability and Biosafety of human epidermal stem cell for wound repair: Preclinical evaluation

Xiaohong Zhao^1^, Xue Li^1^, Ying Wang^1^, Yicheng Guo^1^, Yong Huang^1^, Dalun Lv^2^, [Mingxing Lei](https://pubmed.ncbi.nlm.nih.gov/?sort=date&size=200&term=Lei+M&cauthor_id=28798065)^3^, Shicang Yu^4^, Gaoxing Luo^1a^, and Rixing Zhan^1a^

^1^ Institute of Burn Research, State Key Laboratory of Trauma, Burn and Combined Injury, Southwest Hospital, the Third Military Medical University (Army Medical University), Chongqing 400038, China

^2^ Department of Burn and Plastic Surgery, the First Affiliated Hospital of Wannan Medical College, Wuhu 241001, Anhui, China.

^3^ “111” Project Laboratory of Biomechanics and Tissue Repair, College of Bioengineering, Chongqing University, Chongqing, 400044, China.

^4^ Stem Cell and Regenerative Medicine, Southwest Hospital, the Third Military Medical University (Army Medical University), Chongqing, 400038, China.

^a^: Corresponding author: Rixing Zhan, Email: zhanrixing@sina.com

^a^: Corresponding author: Gaoxing Luo, Email: logxw@hotmail.com

**Materials and Methods**

**1.** **CM-Dil marks EpiSCs**

After the cells were fused to 80%, the cells were collected into the 15ml centrifuge tube, centrifuged for by 800 rpm/min 5 min and the supernatant was removed. The cells were re-suspended with 1 ml PBS and added 2 μl CM-Dil to the solution, then incubate at 37℃ for 5 min, and then incubate at 4℃ for 15 min. Finally, washed with PBS for two times.

**2. Senescence assay**

The cell senescence assay by β-Galactosidase staining kit (Beyotime). The experiment method was carried out according to the instructions. The P4, P8, and P9 EpiSCs were inoculated in a 6-well plate for 3 days, then the medium was absorbed and was washed once with PBS. And then added 1 ml β-galactosidase staining fixation solution to 6-well plate and fixed at room temperature for 15 min. The cell fixation solution was removed and the cells were washed with PBS for 3 min each time. Next, absorbed PBS and added 1 ml dyeing solution to each hole. Incubated at 37℃ overnight, and then observed and recorded under an ordinary optical microscope.

**3. Transcriptome analysis**

For transcriptome analysis, P1, P4, P8 and P9 EpiSCs were incubated in RNAiso Plus (Takara) at a concentration of 1🞨10^6^ cells/ml and stored at -80℃. Three experimental replicates per group were performed. All samples were transported to the Genomics Institute on dry ice for the transcriptome study (Sichuan Panomic Biotechnology Co., Ltd). mRNA was enriched using oligo (dT) magnetic beads and fragmented into short fragments using fragmentation buffer. cDNA libraries were produced and qualified using an Agilent 2100 Bioanalyzer and an ABI Step One Plus Real-Time PCR System. Primary raw reads produced by HiSeq 4000 (Illumina) were qualified and filtered to obtain clean reads. The Pearson correlation coefficients was based on all gene expression levels. A heatmap analysis of gene expression levels was created based on the averaged fragments per kilobase of exon per million fragments mapped (FPKM) values of genes in P1, P4, P8, and P9 EpiSCs. Genes with a fold change in expression>1 and adjusted *P* value≤ 0.001 were considered to be differentially expressed genes (DEGs). the selected DEGs related to tumorigenicity, proliferation and aging were analyzed.

**Figures S1 to S4**

**
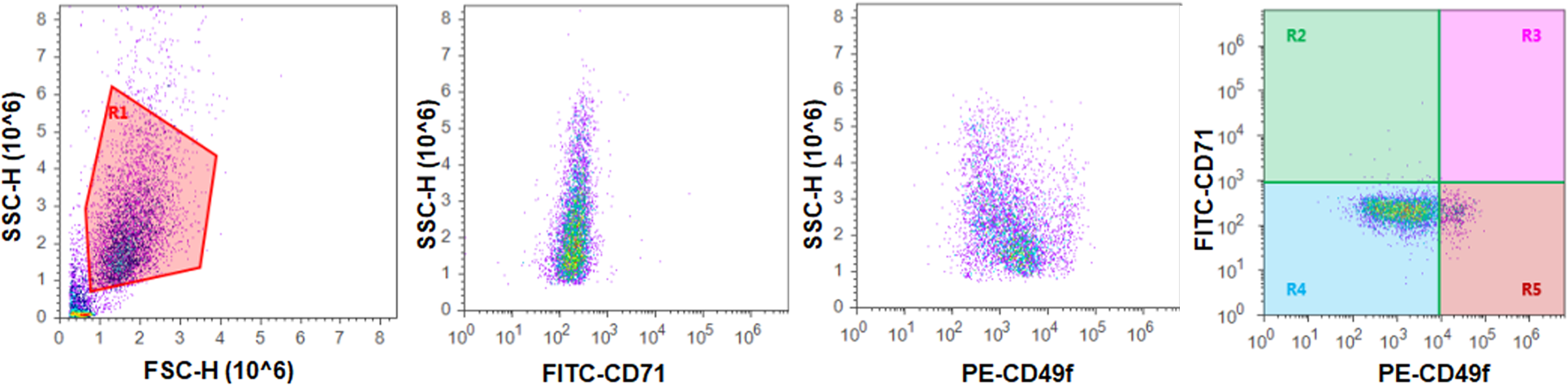
**

**Fig. S1. Identification of EpiSCs from human prepuce.** Flow detection of cell suspension from the epidermis. R5 represents the ratio of high expression CD49f and low expression CD71 was approximately 9%.


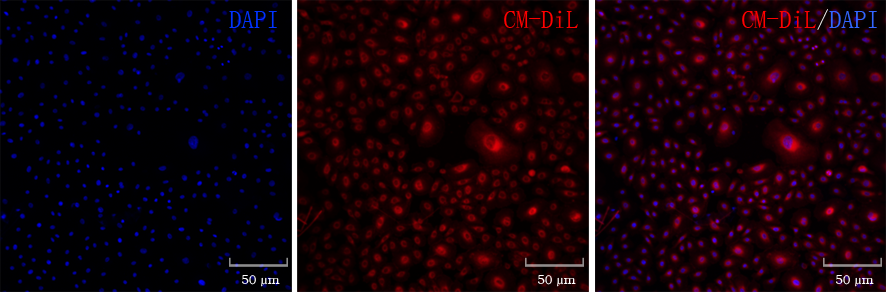


**Fig. S2. EpiSCs (P4) were stained with CM-Dil.** Blue was DAPI, red was CM-DiL (under 20× confocal microscopy).

**
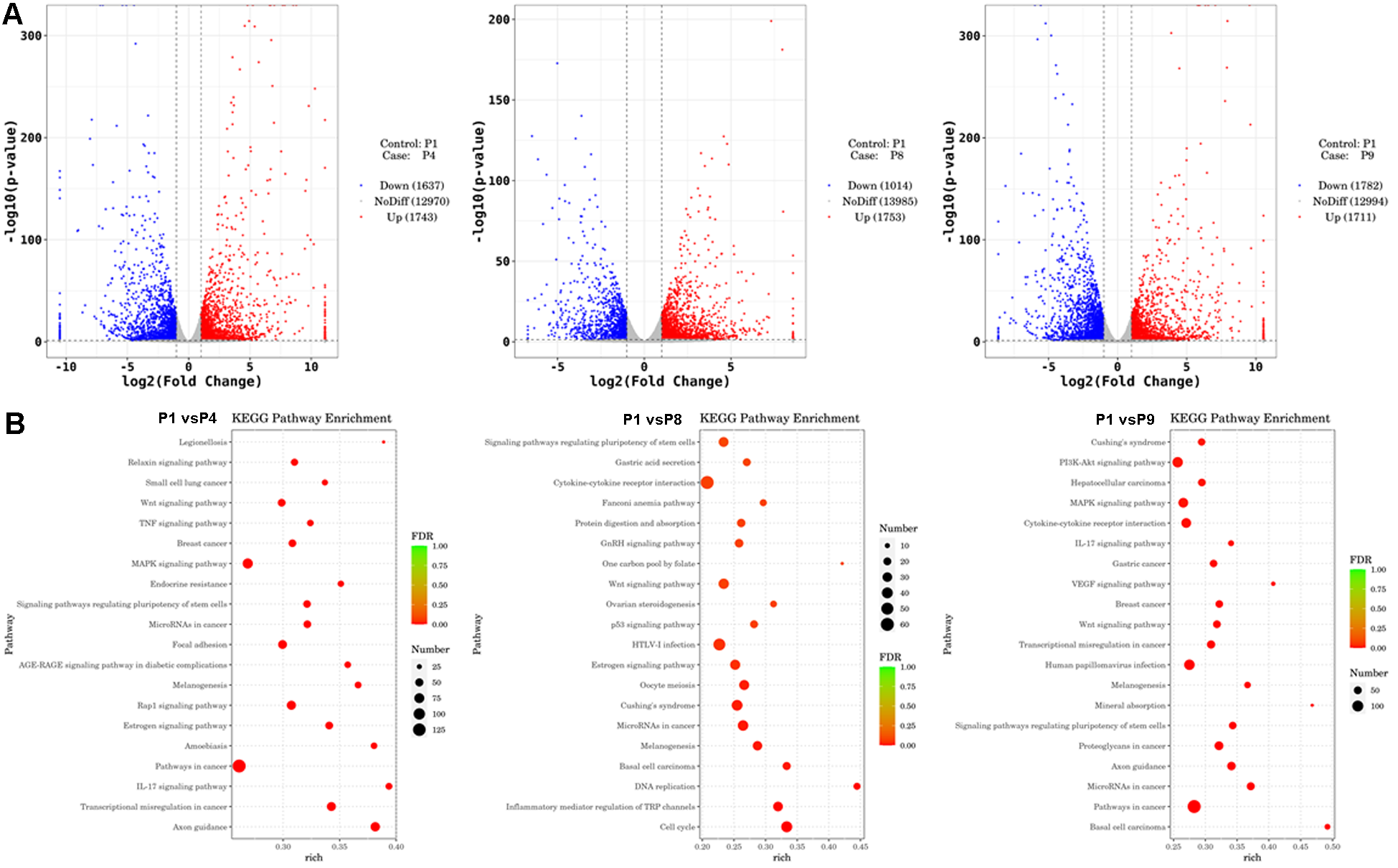
**

**Fig. S3. The** **mRNA sequencing results of EpiSCs.** (A) Relationship between up-regulated and down-regulated genes of DEGs in different groups. (B) KEGG functional classification of the DEGs.


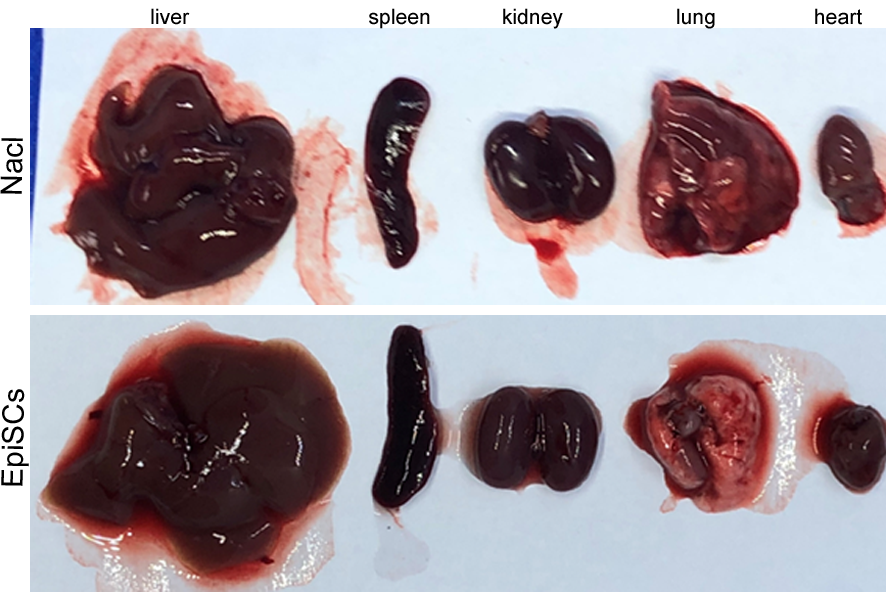


**Fig. S4. The organs of the NaCl group and the EpiSC group.** Representative samples of heart, liver, spleen, lung and kidney of nude mice in NaCl group and in EpiSC group after subcutaneous injection for 4 months.

**Tables S1**

**Table S1. The proliferation curve of EpiSCs**

| Days | Cell passage  number | | Cumulative cells (10^6^) | Cumulative  cell’s area (cm^2^) | Transplantable total body surface area (%) |
| --- | --- | --- | --- | --- | --- |
| 0 | P0 | 5.00 ± 0.00 | | 50.00 ± 0.00 | 0.33 ± 0.00 |
| 7±2 | P0 | 1.80 ± 0.16 | | 50.00 ± 0.00 | 0.33 ± 0.00 |
| 10±2 | P1 | 6.47 ± 0.77 | | 275.00 ± 35.36 | 1.83 ± 0.24 |
| 15±2 | P2 | 21.30 ± 2.43 | | 925.00 ± 127.48 | 6.17 ± 0.85 |
| 20±2 | P3 | 73.93 ± 7.15 | | 3175.00 ± 348.21 | 21.17 ± 2.32 |
| 25±2 | P4 | 254.77 ± 19.05 | | 11050.00 ± 1075.29 | 73.67 ± 7.17 |
| 30±2 | P5 | 880.20 ± 47.08 | | 38150.00 ± 2820.46 | 254.33 ± 18.80 |
| 35±2 | P6 | | 2990.40 ± 188.44 | | --- | | | 130700.00 ± 7813.77 | 871.33 ± 52.09 |
| 40±2 | P7 | | 9864.35 ± 554.24 | | --- | | | 443825.00 ± 22994.27 | 2958.83 ± 153.30 |
| 45±2 | P8 | | 31913.79 ± 2323.04 | | --- | | | 1464000.00 ± 73515.41 | 9760.00 ± 490.10 |

**Note.** Isolated and cultured prepuce EpiSCs from aged 6, 14 and 18 years old. The area of each prepuce was about 1 cm^2^. The cumulative cell area was calculated according to the number of flasks that could be inoculated after cell passage. The proportion of transplantable wound area was calculated according to the cumulative cultured cell area (human total body surface area was 1.5 m^2^). Results were expressed as means ± standard deviation (n=3)

**Quality control data**

**1. Research data on the quality of EpiSCs**

Table S2. Items and standards for testing the quality of EpiSCs

| Test items |  | Detection method | standard |
| --- | --- | --- | --- |
| Cell identification | Cell morphology | Inverted microscope observation | 🗸 |
|  | Specific marker | Flow cytometry | 🗸 |
|  | Cell karyotype | G-band staining method | 🗸 |
| Mycoplasma |  | Fluorescence quantitative PCR | Negative |
| Endotoxin |  | Gel method | <0.5EU/mL |
| Bacteria, fungi |  | Fully automatic microbial culture  Membrane filtration method | Negative |
| Extracellular and extracellular viral factors | TP、HIV、HBV、HCV、HCMV、EBV、HPV | ELISA | Negative |
| Residual components of culture medium | BSA | ELISA | ≤50ng/mL |

**2. Detection of bacteria and fungi**

The cell supernatant samples were collected and double sets of bacterial and fungal examination systems were used to ensure the asepsis of the culture system. In the membrane filtration method, according to the requirements of Chinese Pharmacopoeia 2015 (General principles 1100), the samples were added to thioglycolate fluid medium (culture of anaerobes and aerobes, temperature 30-35℃) and liquid culture medium of casein-soy peptone (culture fungi and aerobes, temperature 20-25℃). It should be negative for 14 days. At the same time, using the fully automatic microbial culture system, the samples were added to aerobic culture flask (BPA) and anaerobic culture flask (BPN) respectively. When BPA and BPN were put into the automatic microbial culture detection system and cultured at 37 ℃ for 7 days, the results should be negative.

**3. Mycoplasma detection**

The supernatant of cell culture was taken and mycoplasma was detected by photometer. The principle was that some specific enzymes are produced after mycoplasma cleavage in the sample. The enzyme reacts with the substrate in the mycoplasma detection kit to catalyze the conversion of ADP to ATP. The presence or absence of mycoplasma can be known by detecting ATP levels before and after the addition of substrates. If mycoplasma enzymes react with their specific substrates, it can lead to an increase in ATP levels. The increased ATP can be detected by bioluminescence reaction, and the intensity of light emitted is linearly correlated with the content of ATP, which can be detected by photometer.

**4. Endotoxin test**

According to the requirements of Pharmacopoeia 2015 (General principles 1143), the supernatant of cell culture was taken to detect the content of endotoxin by gel limit method, that is, the method of limited detection of endotoxin by the principle of agglutination reaction between Limulus amebocyte lysate and endotoxin. According to the formula in Pharmacopoeia, the limit of endotoxin, namely<0.5 EU/mL, was determined, then the maximum and most effective dilution multiple of the sample was calculated, and the substances in the culture supernatant which could not produce Limulus amebocyte lysate were determined by interference test. The sensitivity check test of Limulus amebocyte lysate was carried out each time the use of the new batch number of Limulus reagent or any change in the experimental conditions that might affect the results.

**5. Detection of intracellular and extracellular viral factors**

According to the guidelines for quality Control of Stem Cell preparations and preclinical Research (trial), it is necessary to detect TP, HIV, HBV, HCV, HCMV, EBV and HPV virus in epidermal donors.

**Colloidal gold method**

The cell supernatant samples were taken and the colloidal gold kit was used to detect the above viruses. The principle is based on colloidal gold immune technology and chromatography principle, when detecting positive samples, the virus antibody in the sample forms a complex with colloidal gold labeled antigen, because the chromatographic complex moves forward along the strip, when passing through the detection line, it combines with the pre-coated recombinant virus antigen to form a double antigen sandwich and agglomerates to show color, which is judged to be positive. If the sample does not contain virus antibodies, the detection area will not form a red line and will be judged to be negative. This method is simple and rapid, and can be used as a preliminary judgment, and then checked by the third-party fluorescence quantitative PCR method in the later stage.

**Fluorescence quantitative PCR**

The principle is based on TaqMan probe real-time fluorescence PCR technology, which is suitable for the detection of viral DNA in human clinical serum or plasma samples. TaqMan fluorescence probe detection technology combined with PCR amplification method was used to detect the nucleic acid in the sample. The conserved region of the virus was selected as the detection target gene. The quantitative detection of nucleic acid in the sample was realized through the change of fluorescence signal in the process of PCR and the change of fluorescence signal value amplified by DNA standard sample. Using exogenous internal parameters, we can monitor and avoid "false negative" results caused by PCR inhibitors or improper operation in the sample.

**6. Third-party testing (Microbiology Laboratory of Burn Research Institute)**

In addition to the self-test, the microbiology laboratory of the Institute of Burn Research should be retested as required to detect the quality of EpiSCs (including viral factors (TP, HIV, HBV, HCV, HCMV, EBV, HPV), aseptic test, mycoplasma test, endotoxin test and bovine serum albumin residue test.

**7. Cell activity detection**

Three batches of cells were randomly selected to detect the cell viability before and after frozen storage. The experimental results showed that the cells could express the viability requirements after cryopreservation.

**
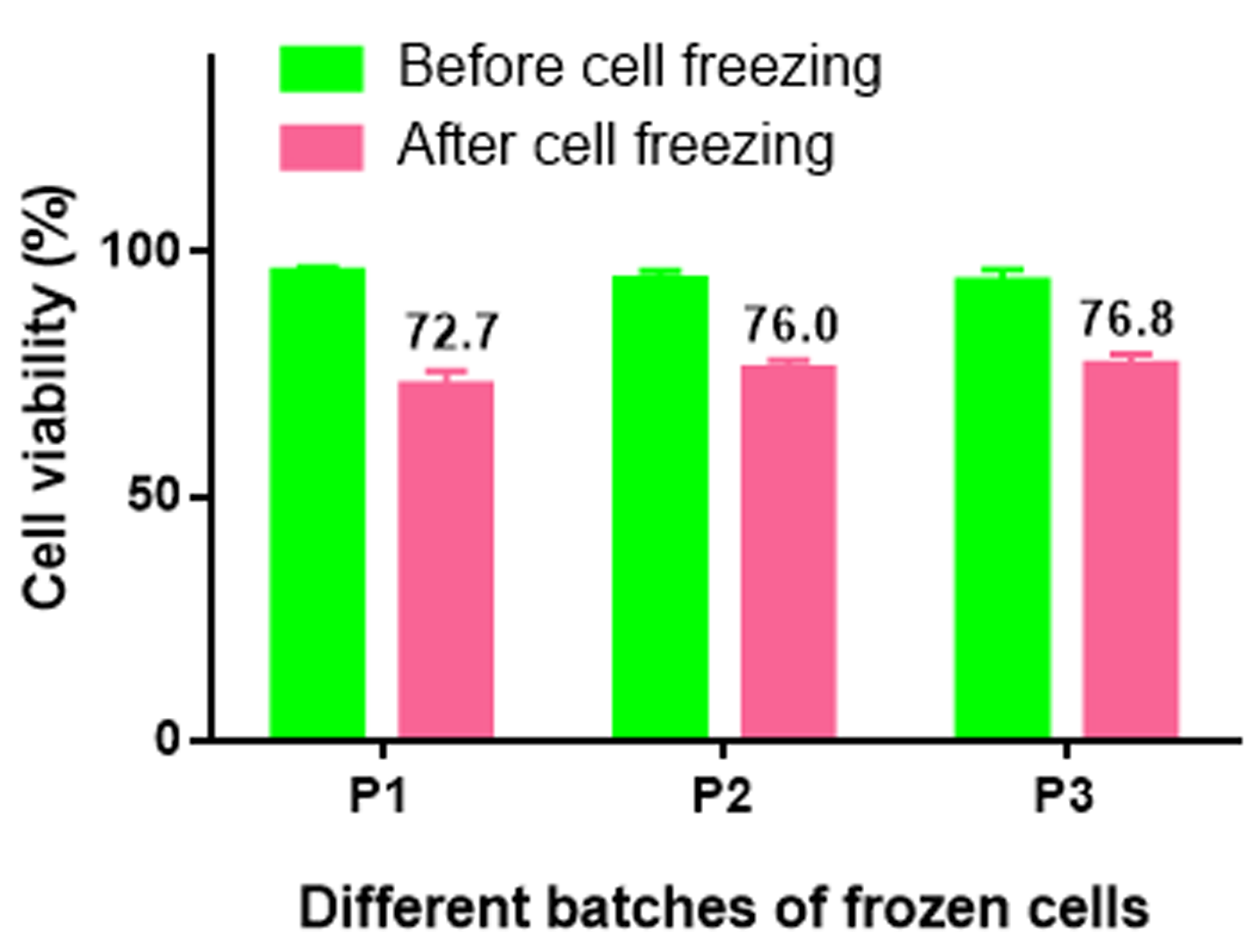
**

**Fig. S5. Statistical results of cell viability before and after cryopreservation**

**8. High temperature test**

The EpiSCs injection was placed in a 37℃ incubator for 6 hours. Samples were taken at the different time to investigate the character (number of flocs), cell number, cell viability and phenotypic detection. The detection methods are observation, counting and flow detection. The results showed that sustained high temperature led to a decrease in the vitality and number of epidermal cells.


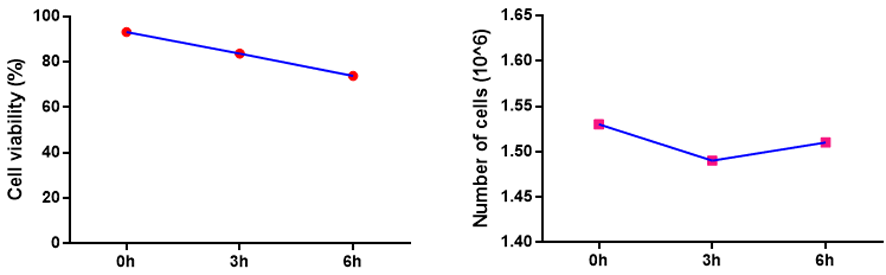


**Fig. S6. Effect of high temperature on epidermal cells**

**9. Low temperature test**

Drugs prone to biological phase separation, viscosity reduction, precipitation or aggregation need to be tested by thermal cycle experiments to verify their stability in the process of transportation or use. The epidermal cell injection was used for thermal cycling experiment for three times, each cycle should be at 2-8 ℃ for 1 hour, and then accelerated at 25 ℃ for 1 hour. The results showed that thermal cycle had little effect on the maintenance of epidermal cell state.

**
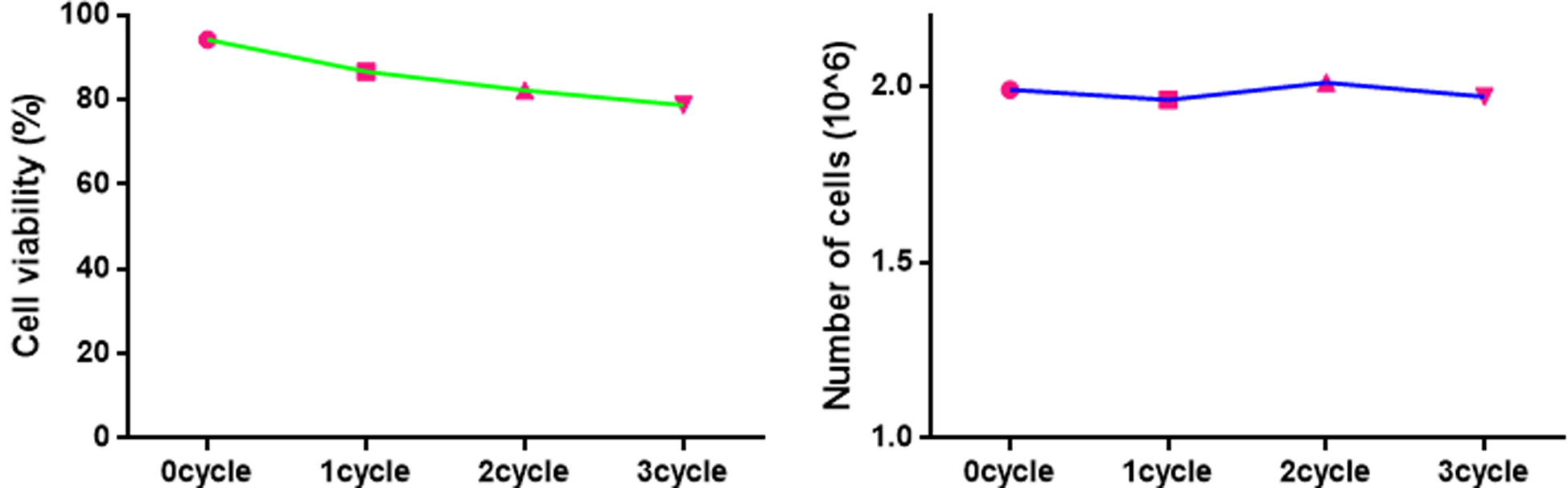
**

**Fig. S7. Effect of low temperature on epidermal cells**

**10.** **Long-term test**

The prepared epidermal cell injection was placed in a medical freezer with a temperature of 6℃ ±2℃ and samples were taken every 6 hours to observe the changes of cell number, cell viability and cell phenotype. The results showed that the activity of epidermal cell suspension was good within 6 hours, and then decreased sharply.

**
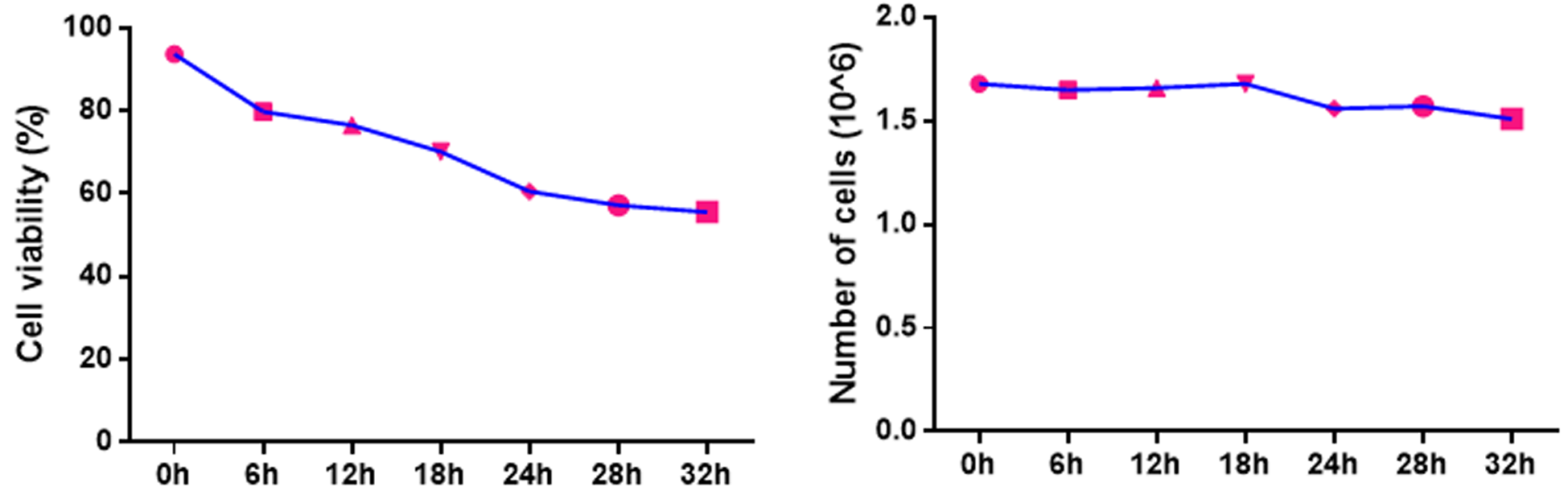
**

**Fig. S8. Effect of long-term experiment on epidermal cells**

**11.** **Do epidermal stem cells express mesenchymal stem cell markers (CD73, CD90, CD105)?**


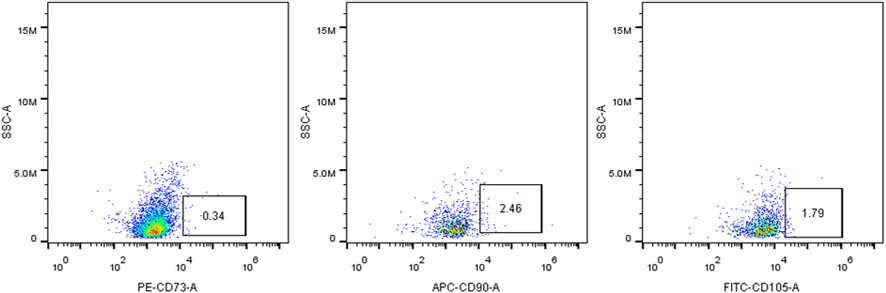


**Fig. S9.** Detection of CD73, CD90, and CD105 expression in EpiSCs (P8) by flow cytometry. There was almost no expression of CD73, CD90, and CD105.
